# Treatment outcome and long-term follow-up of central nervous system germ cell tumor using upfront chemotherapy with subsequent photon or proton radiation therapy: a single tertiary center experience of 127 patients

**DOI:** 10.1186/s12885-020-07484-y

**Published:** 2020-10-09

**Authors:** Kyung Taek Hong, Da Hye Lee, Bo Kyung Kim, Hong Yul An, Jung Yoon Choi, Ji Hoon Phi, Jung-Eun Cheon, Hyoung Jin Kang, Seung-Ki Kim, Joo-Young Kim, Sung-Hye Park, Il Han Kim, Hee Young Shin

**Affiliations:** 1 Departments of Pediatrics, Seoul National University College of Medicine, Seoul, Republic of Korea; 2 Division of Pediatrics, Seoul National University Children’s Hospital, Seoul, Republic of Korea; 3 Seoul National University Cancer Research Institute, Seoul, Republic of Korea; 4 Division of Pediatric Neurosurgery, Seoul National University Children’s Hospital, Seoul, Republic of Korea; 5 Department of Neurosurgery, Seoul National University College of Medicine, Seoul, Republic of Korea; 6 Department of Radiology, Seoul National University College of Medicine, Seoul, Republic of Korea; 7 Proton Therapy Center, Research Institute and Hospital, National Cancer Center, Goyang, Republic of Korea; 8 Department of Pathology, Seoul National University College of Medicine, Seoul, Republic of Korea; 9 Department of Radiation Oncology, Seoul National University College of Medicine, Seoul, Republic of Korea; 10 Department of Pediatrics, Seoul National University College of Medicine, Seoul National University Cancer Research Institute, 101, Daehak-ro, Jongno-gu, Seoul, 03080 Republic of Korea

**Keywords:** Germ cell tumor, Central nervous system, Intracranial, Secondary malignant neoplasm, Proton therapy

## Abstract

**Background:**

Central nervous system germ cell tumors (CNS GCTs) are a heterogeneous group of brain tumors, which are more common in Asian countries. There have been different therapeutic strategies in treating germinoma and non-germinomatous germ cell tumors (NGGCT), depending on prognosis. Moreover, long-term follow up should be emphasized due to higher late complication rates. Here, we investigated long-term outcomes and complication profiles of 127 CNS GCT patients who received uniform upfront chemotherapy.

**Methods:**

We retrospectively evaluated outcomes of CNS GCT patients treated in Seoul National University Children’s Hospital from August 2004 to April 2019. Patients were classified as low risk (LR) or high risk (HR) based on pathologic diagnosis and tumor markers. Most patients received upfront systemic chemotherapy with carboplatin, cyclophosphamide, etoposide, and/or bleomycin, followed by either proton or photon radiation therapy according to patients’ choice.

**Results:**

The median age at diagnosis was 11.9 (range, 3.8–25.1) years, and 54.3% of patients were LR. Photon and proton radiation therapy were administered to 73.2 and 25.2% of patients, respectively. In both LR and HR groups, there were no significant differences in survival between photon and proton radiation therapy. The 10-year relapse incidences were 9.3 and 5.6% in the LR and HR groups, respectively. All recurrences, except one, were local relapse. Six secondary malignancies occurred; the 10-year incidences of secondary malignancy were 2.2 and 7.6% in the LR and HR groups, respectively. The 10-year overall survival rates were 98.3 ± 1.7 and 91.8 ± 3.9% in the LR and HR groups, respectively. In a subgroup analysis of HR group, pathologically diagnosed NGGCT patients (*n* = 20) showed worse 10-year EFS (65.9 ± 11.9%, *p* < 0.001) and OS (77.9 ± 9.8%, *p* = 0.024) rates compared to other HR patients who were not pathologically diagnosed or were confirmed as germinoma with elevated tumor markers. All mortalities were related to disease progression or secondary malignancy.

**Conclusion:**

The strategy of treating CNS GCTs with upfront chemotherapy according to risk groups resulted in good clinical outcomes and acceptable relapse incidence. However, further modification in the definition of the HR group is needed to reduce long-term complications.

## Background

Childhood central nervous system germ cell tumors (CNS GCTs) are a heterogeneous group of brain tumors that are more common in Asian countries than in Western countries. In South Korea, CNS GCTs account for 16% of all primary CNS tumors in individuals aged below 15 years, whereas in Western countries, they approximately account for 3–4% [1].

Generally, they are classified into germinoma and non-germinomatous germ cell tumors (NGGCTs). Germinoma is known to be curable with craniospinal irradiation (CSI) and local boost radiation therapy alone. However, the use of neoadjuvant or upfront chemotherapy allows for reduced radiation therapy doses and volumes, which subsequently reduces long-term radiation therapy-related adverse effects. Whole ventricle irradiation followed by a boost to the primary site is generally accepted for localized germinomas [2, 3], whereas CSI with local boost could be administered for metastatic germinomas [4]. Although localized germinoma shows good outcomes, chemotherapy alone or focal irradiation without whole ventricle is not an effective treatment option [5–7].

For NGGCT, various upfront chemotherapy regimens with CSI have been used. In Children’s Oncology Group trial, neoadjuvant chemotherapy using carboplatin, etoposide, and ifosfamide and subsequent radiation therapy with CSI (36 Gy) and local boost (54 Gy) resulted in a 5-year event-free survival (EFS) rate of 84 ± 4% and 5-year overall survival (OS) rate of 93 ± 3% [8]. For nonmetastatic NGGCT, focal irradiation was only used with neoadjuvant chemotherapy using cisplatin, etoposide, and ifosfamide in SIOP-CNS-GCT-96 trial. The 5-year EFS and OS rates for localized NGGCT were 72 ± 4 and 82 ± 3%, respectively [9].

Given that CNS GCTs have relatively better outcomes than other pediatric brain tumors, the need to reduce long-term complications, while preserving clinical outcomes, has been emphasized. Here, we report the long-term outcomes and complication profiles of 127 patients with CNS GCTs in a single tertiary center, where uniform upfront chemotherapy had been administered.

## Methods

### Patient characteristics

**Fig. 1:**
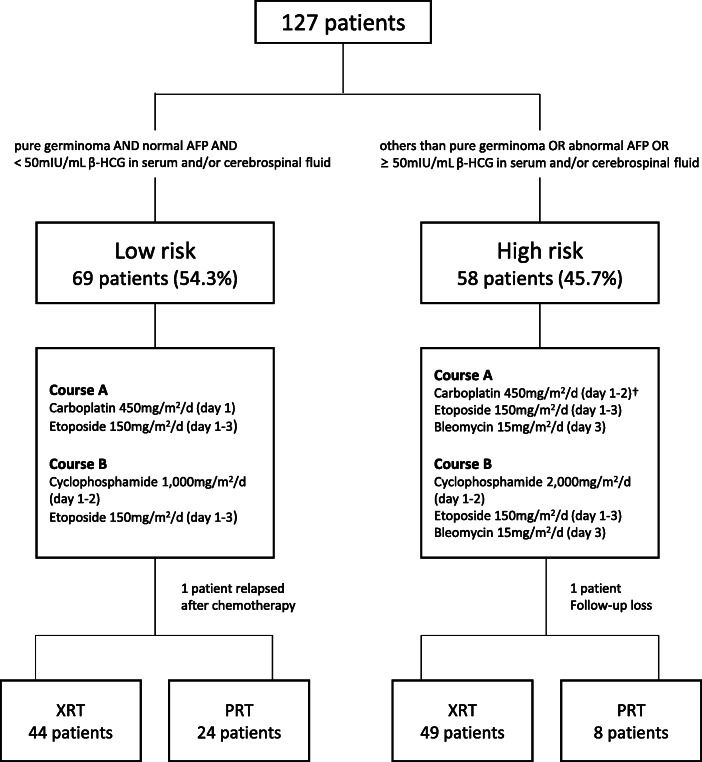
Overall treatment scheme and classification of patients. Upfront chemotherapy alternating between courses A and B, and a total of 4 cycles of chemotherapy (A-B-A-B) were administered. Subsequent radiation therapy was administered using photon (XRT) or proton (PRT) therapy according to the patients’ choice. **†**Carboplatin 450 mg/m^2^ was administered on day 1 until 2008, whereas the same dose of carboplatin was administered on days 1–2 from 2009

We retrospectively reviewed the data of 127 patients diagnosed with CNS GCTs at Seoul National University Children’s Hospital from August 2004 to April 2019. From August 2004, current upfront chemotherapy regimen has been used. Before receiving upfront chemotherapy, patients were classified as low risk (LR) or high risk (HR) according to pathologic diagnosis and tumor markers. LR was defined as patients with pure germinoma with normal α-fetoprotein (AFP) and < 50 mIU/mL β-human chorionic gonadotropin (β-HCG) levels in the serum and/or cerebrospinal fluid; the remaining patients were classified as HR. The classification of all patients and the overall treatment scheme are shown in Fig. 1. The median follow-up periods were 8.4 years (range, 0.3–14.1) in the LR group and 8.3 years (range, 0.2–14.2) in the HR group, respectively.

The co-primary outcomes of this study were EFS and OS rates of each risk group. Secondary outcomes were relapse incidence, late complications, and the cumulative incidence of secondary malignant neoplasms (SMNs). The Institutional Review Board approved the procedure of reviewing medical records, and obtaining consent was waived (H-1906-155-1045).

### Upfront chemotherapy and radiotherapy

The majority of patients received upfront systemic chemotherapy first. All medications were administered intravenously once daily. For the LR group, course A comprised carboplatin 450 mg/m^2^ on day 1 and etoposide 150 mg/m^2^ on days 1–3, and course B included cyclophosphamide 1000 mg/m^2^ on days 1–2 and etoposide 150 mg/m^2^ on days 1–3. For the HR group, course A comprised carboplatin 450 mg/m^2^ on day 1 until 2008, whereas the same dose of carboplatin was administered on days 1–2 from 2009 in order to increase the therapeutic effect, etoposide 150 mg/m^2^ was administered on days 1–3 and bleomycin 15 mg/m^2^ on day 3. Course B for the HR group included cyclophosphamide 2000 mg/m^2^ on days 1–2, etoposide 150 mg/m^2^ on days 1–3, and bleomycin 15 mg/m^2^ on day 3. Alternating between courses A and B, a total of 4 chemotherapy treatments were performed with a 3-week interval. After the final chemotherapy cycle (second course B), subsequent photon radiation therapy (XRT; Varian, Palo Alto, CA) was conducted in our institution. If the patient wanted to receive proton radiation therapy (PRT; IBA, Louvain-La-Neuve, Belgium), it was conducted at another institution (National Cancer Center). The range of radiation therapy was described as CSI, whole-brain, whole ventricle and primary sites. CSI, whole-brain or whole ventricle irradiation were applied mutually exclusively.

### Statistical analysis

Categorical and continuous variables were compared using Pearson’s chi-square test or Fisher’s exact test and the Student’s t-test, respectively. The incidence rates of relapse and SMNs were calculated using a cumulative incidence function. Progression-free survival (PFS), EFS, and OS were analyzed using the Kaplan–Meier method. Events were defined as death, relapse, or SMNs. Differences in survival rates were investigated using a log-rank test. *p* < 0.05 was considered statistically significant. Statistical analyses were conducted using R version 3.2.2 (www.r-project.org) and SPSS 23.0 (IBM-SPSS, Armonk, NY).

## Results

### Patient characteristics and tumor markers

Among the 127 cases, 69 patients (54.3%) were LR, and 58 patients (45.7%) were HR. The median age at diagnosis of LR and HR groups were 11.8 (range, 3.8–18.9) and 12.2 (4.5–25.1) years, respectively (Table 1). The initial presentation and tumor location were not different between both groups. Of all patients, pathologic diagnosis was confirmed in 100 patients (78.7%), of whom 80 patients had germinoma. The remaining 27 patients were diagnosed by the results of tumor markers and typical image findings. In LR group (*n* = 69), 66 patients were germinoma and 3 were not pathologically confirmed. Because the tumor markers were normal, the latter 3 patients needed a biopsy. However the risk was high for biopsy and the symptoms progressed rapidly, thus biopsy was omitted. In HR group (*n* = 58), 14 patients were germinoma and 24 were not pathologically confirmed. Of the remaining 20 pathologic-confirmed HR patients, 14 were mixed germ cell tumors, 5 were immature teratomas, and 1 was a choriocarcinoma.

**Table 1:** Characteristics of all patients (*N* = 127)

Characteristics	Low risk (*n* = 69)	High risk (*n* = 58)	*p* value
Sex			0.852
Male	55 (79.7%)	47 (81.0%)	
Female	16 (20.3%)	11 (19.0%)	
Age, median (range)	11.8 (3.8–18.9)	12.2 (4.5–25.1)	0.606
Initial presentation			
Headache	35 (50.7%)	35 (60.3%)	0.278
Vomiting	28 (40.6%)	28 (48.3%)	0.384
Endocrine problems	21 (30.4%)	18 (31.0%)	0.942
Visual problems	19 (27.9%)	19 (32.8%)	0.557
Neurologic symptoms	20 (29.0%)	14 (24.1%)	0.539
Tumor location			0.223
Pineal	24 (34.8%)	28 (47.5%)	
Sellar or suprasellar	14 (20.3%)	13 (22.0%)	
Thalamus or basal ganglia	17 (24.6%)	7 (11.9%)	
Ventricle	1 (1.4%)	1 (1.7%)	
Bifocal	10 (14.5%)	7 (11.9%)	
Multifocal	3 (4.3%)	1 (1.7%)	
Brain stem	0 (0.0%)	1 (1.7%)	
Pathologic diagnosis			< 0.001
Germinoma	66 (95.7%)	14 (23.7%)	
Immature teratoma	0 (0.0%)	5 (8.5%)	
Choriocarcinoma	0 (0.0%)	1 (1.7%)	
Mixed	0 (0.0%)	14 (23.7%)	
Unknown	3 (4.3%)	24 (40.7%)	
Tumor seeding on MRI, Yes	7 (10.1%)	7 (12.3%)	0.704
Initial hydrocephalus, Yes	39 (56.5%)	30 (51.7%)	0.589
ETV, Yes	28 (40.6%)	23 (39.7%)	0.916
EVD, Yes	24 (34.8%)	14 (24.1%)	0.192
Initial EVD and subsequent VP shunt insertion, Yes	5 (7.2%)	6 (10.3%)	0.536
Direct VP shunt insertion, Yes	5 (7.2%)	4 (6.9%)	0.939
Operation due to growing teratoma, Yes	2 (2.9%)	12 (20.7%)	0.001
Follow-up year, median (range)	8.4 (0.3–14.1)	8.3 (0.2–14.2)	0.706

*NGGCT* nongerminomatous germ cell tumor, *MRI* Magnetic resonance imaging, *ETV* endoscopic third ventriculostomy, *EVD* extraventricular drainage, *VP* ventriculoperitoneal

Between both groups, the frequency of initial hydrocephalus, endoscopic third ventriculostomy, and extraventricular drainage (EVD) was similar. Three patients with inserted EVD received the first cycle of chemotherapy after which EVD could be successfully removed 2 or 3 weeks after the initiation of chemotherapy without any complications or subsequent ventriculoperitoneal shunt insertion. Due to growing teratoma syndrome, 2 patients of LR group and 12 patients of HR group were operated. The pathology of 9 patients were mature teratoma, while the remaining 3 patients had immature teratoma. The median follow-up time of LR and HR patients were 8.4 and 8.3 years, respectively (Table 1).

Serum/cerebrospinal fluid (CSF)-AFP and β-HCG levels were examined in all available patients (*n* = 124). Only serum tumor markers were evaluated in the remaining 3 patients. The median serum and CSF-AFP levels were 1.3 (range, 0–16.1) ng/mL and 0 (range, 0–4.0) ng/mL, respectively, in the LR groups and 11.1 (range, 0–5470) ng/mL and 0.7 (range, 0–1616) ng/mL, respectively, in the HR groups. Moreover, the median serum and CSF-β-HCG levels were 0.4 (range, 0–23.0) mIU/mL and 1.9 (range, 0–43.5) mIU/mL, respectively, in the LR groups and 18.5 (range, 0–26,760) mIU/mL and 87.7 (range, 0–70,420) mIU/mL, respectively, in the HR groups.

### Radiotherapy

After 4 cycles of upfront chemotherapy, subsequent radiotherapy was given if the tumor did not progress. According to patients’ choice, XRT at our institution or PRT at another institution (National Cancer Center) were carried out. Most of the reasons for choosing PRT were expectations that it could reduce long-term complications. The area and dose of radiation were heterogeneous due to the differences in responses to upfront chemotherapy and the presence of tumor seeding. Generally, CSI was conducted in patients with germinoma with leptomeningeal or ventricular seeding, or those diagnosed with NGGCT. However, radiotherapy could be adapted to the response to upfront chemotherapy.

Of the 68 patients in the LR group, 44 (64.7%) and 24 (35.3%) patients received XRT and PRT, respectively. One patient was not included because the tumor recurred after chemotherapy. Craniospinal irradiation (CSI, median dose 23.4 Gy, range 19.8–36.0), whole-brain irradiation (median dose 30.6 Gy, range 23.4–39.6), and whole ventricle irradiation (median dose 19.8 Gy, range 16.2–45.0) were performed in 21 (30.9%), 2 (2.9%), and 43 (65.2%) patients, respectively. Two patients were irradiated only on the primary site. The median total dose of primary site in the LR group was 40.4 (range, 27.0–59.2) Gy. The main reasons for CSI were either seeding on MRI (magnetic resonance imaging), multifocal initial lesions, or relatively poor response to upfront chemotherapy.

Of the 57 patients in the HR group, 49 (86.0%) and 8 (14.0%) patients received XRT and PRT, respectively. Forty-six (80.7%) patients received CSI (median dose 23.4 Gy, range 18.0–36.0), while 1 (1.8%) and 10 (17.5%) patients received whole-brain (30.6 Gy) and whole ventricle irradiation (median 23.4 Gy, range 18.0–39.6), respectively. The median total dose of primary site in the HR group was 54.0 Gy (range, 27.0–59.4), which is significantly higher than that of the LR group (40.4 Gy [range, 27.0–59.2], *p* < 0.001). Eleven patients (19.3%) omitted CSI, all of which had no MRI seeding and had good reactions after the initial chemotherapy or preoperative resection of the tumor. Only two of them were pathologically proven to be NGGCT. Moreover, in a subanalysis of the HR group, 14 patients who were pathologically diagnosed as germinoma with elevated tumor markers received less total dose of radiation therapy than the groups who had pathologically confirmed NGGCT or unknown histology (Table 2).

**Table 2:** Radiation field and dose of HR group (*N* = 57^a^)

	Pathologic diagnosis of HR group			*p* value
	Germinoma (*n* = 14)	Not confirmed (*n* = 23)	NGGCT (*n* = 20)	
Number of patients who received CSI	9 (64.3%)	19 (82.6%)	18 (90.0%)	0.181
Median dose of CSI, Gy (range)	23.4 (19.8–25.2)	23.4 (18.0–36.0)	23.4 (19.8–36.0)	0.507
Median dose of total radiation to the primary site, Gy (range)	45.0 (27.0–54.0)	54.0 (30.6–59.4)	54.0 (39.6–54.0)	0.001

*NGGCT* nongerminomatous germ cell tumor, *CSI* craniospinal irradiation, *Gy* gray

^a^Of total 58 patients of HR group, 57 patients were included

### Response and relapse

After the completion of chemotherapy and radiotherapy, 92.8 and 91.2% of the LR and HR groups, respectively, showed complete or partial response by MRI. The 10-year relapse incidences were 9.3 and 5.6% in the LR and HR groups, respectively. Among 5 relapsed patients in the LR group, 4 patients had germinoma of basal ganglia. Two of them received radiation therapy only on the primary site, and none of the patients who received the CSI had relapsed. The relapsed sites were all different from the primary site. Particularly, 1 case of relapse presented in the form of a mass in the biopsy track after the end of upfront chemotherapy. All 3 relapsed patients in the HR group showed local relapse and died of disease progression. None of the patients showed spinal seeding (Table 3).

**Table 3:** Characteristics of relapsed patients

Risk	Number	Primary site	Previous radiotherapy	Relapsed site	Time to relapse (days)	Survival
Low risk	1	Rt. BG	LFRT 39.6Gy (XRT)	Lt. medial temporal lobe	660	DOD
	2	Rt. BG	LFRT 39.6Gy (XRT)	Rt. frontal & anterior temporal lobe	1146	Alive
	3	Rt. BG	WV 19.8Gy + LFRT 19.8Gy (PRT)	Subependymal seeding	885	Alive
	4	Lt. BG	WV 19.8Gy + LFRT 19.8Gy (PRT)	Sellar area	2654	Alive
	5	suprasellar	Only chemotherapy	Biopsy track	102	Alive
High risk	1	3rd ventricle	CSI 23.4Gy + LFRT 30.6Gy (XRT)	Pineal gland	449	DOD
	2	Pineal gland	WV 23.4Gy + LFRT 30.6 (XRT)	Pineal gland	201	DOD
	3	Pineal gland	CSI 23.4Gy + LFRT 30.6Gy (XRT)	Pineal gland	365	DOD

*Rt.* right, *BG* basal ganglia, *LFRT* limited-field radiation therapy, *Gy* gray, *Lt.* left, *DOD* died of disease, *XRT* photon radiation therapy, *WV* whole ventricle, *PRT* proton radiation therapy, *CSI* craniospinal irradiation

### Treatment outcome

**Fig. 2:**
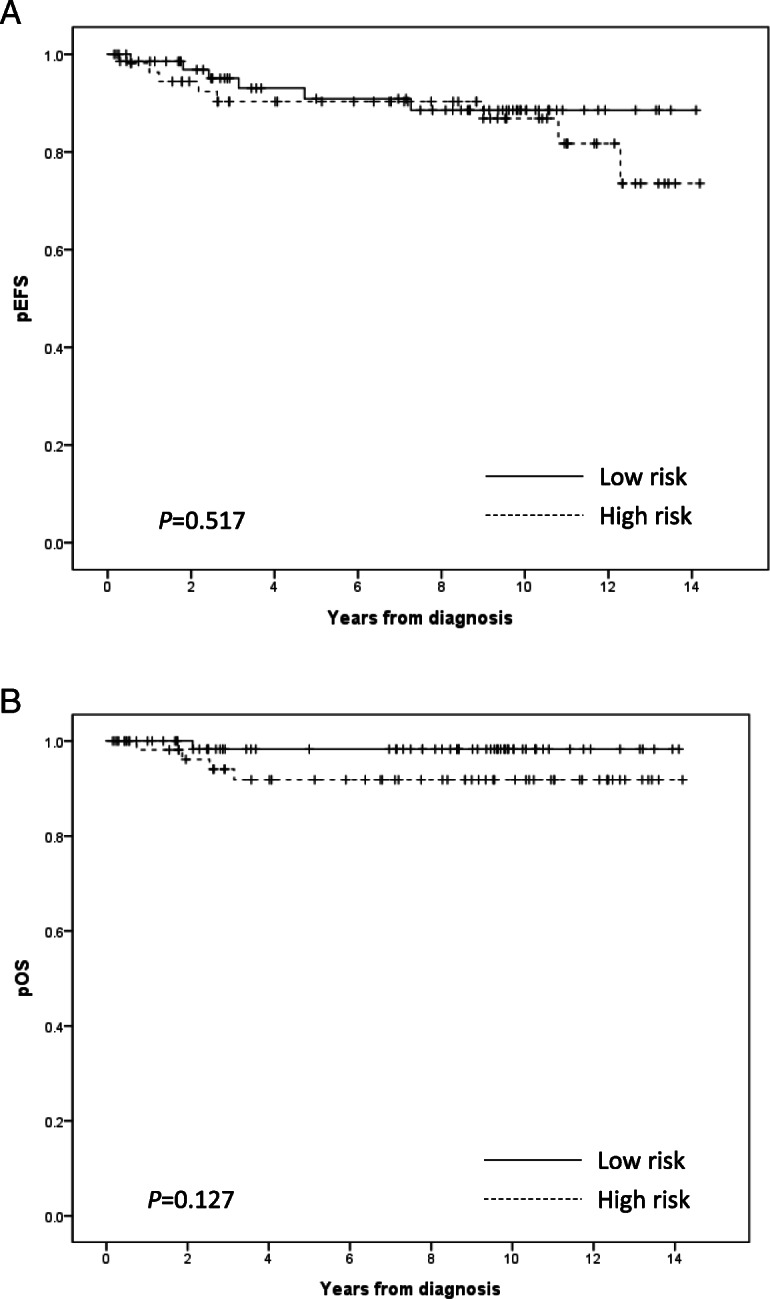
The 10-year event-free survival (EFS) rates in the low risk (LR) and high risk (HR) groups were 88.6 ± 4.5 and 86.8 ± 5.2%, respectively (**a**). Events were defined as death, relapse, or secondary malignant neoplasms. The 10-year overall survival (OS) rates were 98.3 ± 1.7 and 91.8 ± 3.9% in the LR and HR groups, respectively (**b**)

The 10-year EFS and PFS rates were 88.6 ± 4.5 and 90.8 ± 4.0% in the LR group, and 86.8 ± 5.2 and 92.2 ± 3.8% in the HR group, respectively. The 10-year OS rates in the LR and HR groups were 98.3 ± 1.7 and 91.8 ± 3.9%, respectively (Fig. 2A, B). In the LR group, remarkably, basal ganglia germinoma showed lower 10-year EFS rates compared to other parts of germinoma (68.9 ± 13.1% versus 95.2 ± 3.4%, *p* = 0.012).

In the HR group (*n* = 58), the pathologic diagnosis of 14 patients was germinoma, but they were classified into the HR group due to serum or CSF-β-HCG levels being above 50 mIU/mL (*n* = 12), or abnormal AFP levels (*n* = 2). These patients showed 100% EFS and OS rate at 10 years. Moreover, 24 patients who were not pathologically confirmed also had a 100% EFS and OS rate at 10 years. In a subgroup analysis, pathologically diagnosed NGGCT patients (*n* = 20) showed worse 10-year EFS (65.9 ± 11.9%, *p* < 0.001) and OS (77.9 ± 9.8%, *p* = 0.024) rates compared to other groups who were not pathologically diagnosed or were confirmed as germinoma.

### Comparison between XRT and PRT

In our cohort, both XRT and PRT were used, and patients were the ones to decide the type of radiotherapy. In the LR group, the 5-year EFS rate (XRT 91.9 ± 4.5% [*n* = 44] versus PRT 94.1 ± 5.7% [*n* = 24], *p* = 0.457) and OS rate (XRT 97.4 ± 2.5% versus PRT 100%, *p* = 0.485) were similar. In the HR group, only 8 patients received PRT while 49 patients received XRT. Although comparisons are difficult due to the lack of PRT cases, there were no significant differences in the 5-year EFS rate (XRT 91.3 ± 4.2% versus PRT 80.0 ± 17.9%, *p* = 0.605) and OS rate (XRT 93.3 ± 3.8% versus PRT 75 ± 21.7%, *p* = 0.392) between the types of radiotherapy.

### Late complications and secondary malignant neoplasms

**Fig. 3:**
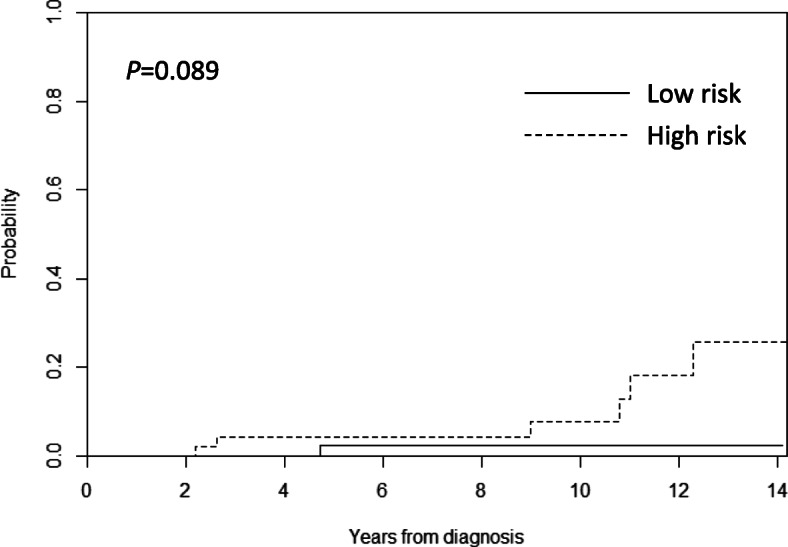
The 10-year cumulative incidences of secondary malignant neoplasms were 2.2 and 7.6% in the low risk (LR) and high risk (HR) groups, respectively

In our cohort, 7 patients experienced secondary neoplasms. Excluding 1 patient with a benign meningioma, 6 patients were diagnosed with SMN, 2 with thyroid carcinomas, 1 with acute lymphoblastic leukemia, 1 with acute myeloid leukemia, 1 with diffuse large B-cell lymphoma, and 1 with atypical meningioma. The 10-year cumulative incidences of SMN were 2.2% in the LR group and 7.6% in the HR group (Fig. 3). However, thyroid carcinoma and meningioma occurred 10 years after the diagnosis of germ cell tumors, which means that long-term follow-up is essential for the surveillance of these types of SMN. A patient who had secondary acute myeloid leukemia died due to fungal pneumonia which was a complication from SMN treatment, while others were treated successfully.

Late complications, including endocrine, neurologic and ophthalmologic problems, are also critical issues. Until the last follow-up, 53.6 and 53.4% of LR and HR patients suffered from endocrine complications, while 18.8 and 15.5% of LR and HR patients showed neurologic complications, such as seizures or severe motor weakness. Moreover, persistent ophthalmologic complications occurred in 19.1 and 8.6% of LR and HR patients. The profile of late complications was not significantly different between the LR and HR groups.

## Discussion

We retrospectively analyzed the treatment outcome of CNS GCTs, treated with homogenous upfront chemotherapy composed of carboplatin, cyclophosphamide, etoposide, and/or bleomycin, and radiotherapy with XRT or PRT. The dose and location of radiotherapy was not equally applied as the decision on the type of radiotherapy rested on the patients. In this study, the 10-year EFS and OS rates were 86.8–88.6 and 91.8–98.3%, respectively, according to the risk group, which is comparable with the rates reported in other studies [3, 8–10]. In particular, this study showed no significant difference in treatment outcomes between LR and HR groups.

In this study, patients were grouped as LR or HR on the basis of tumor markers and pathologic results, which is similar to the classifications used for germinoma and NGGCT in other studies [8, 9]. There were 14 patients in the HR group who were diagnosed with pure germinoma, as determined by surgical biopsy, but showed elevated tumor markers. Their outcome was excellent, with 100% of 10-year EFS and OS rates. The patients with unknown pathology also showed better treatment outcome compared to those who were diagnosed as NGGCT pathologically. Because the rise of tumor markers can mean that there are high-grade germ cell tumors other than geminoma that have not been found in biopsy, they have also received HR chemotherapy. However, our results suggest that only an increase in tumor markers does not necessarily mean a prognosis like NGGCT which has been confirmed by biopsy. Since the number of patients is too small to reach a conclusion, further research is required to elucidate whether histologically proven germinomas with elevated tumor markers could receive a de-escalated treatment. Our findings also suggest that further modification in the definition of the HR group is needed.

The majority of relapse cases of the LR group was basal ganglia germinoma. Because, half of the relapsed patients were only treated with local radiation therapy, it requires attention to interpretation of the bad prognosis of basal ganglia germinoma. Nonetheless, our finding shows the need for a sufficient radiation field in this type of tumor. In our study, heterogeneous radiation therapy was performed, and 30.9% of LR group patients were treated with CSI. There was no recurrence in all of them, but one had SMN. Therefore, considering the good prognosis of the LR group, except for basal ganglia germinoma, the application of CSI needs to be decided more carefully.

Although the number of patients was small, there were no differences in the clinical outcomes between XRT and PRT treated patients. However, given that this was a retrospective analysis, and that the type of radiotherapy was decided by the patients, it is hard to compare the 2 groups in this study. The comparison between XRT and PRT has been consistently reported in pediatric tumors [11]. One study reported no significant differences in treatment outcomes between XRT and PRT for standard risk medulloblastoma [12]. However there are reports that PRT showed better outcomes in terms of toxicity and long-term sequelae [11, 13]. Since many factors could be involved in the selection of PRT [14], further long-term follow-up may be necessary to compare the prognosis and complications between XRT and PRT.

Among long-term complications, SMN is one of the most critical problems in childhood and adolescent cancer survivors [15, 16]. In this study, the 10-year cumulative incidences of SMN in CNS GCTs patients were 2.2% in the LR group and 7.6% in the HR group. The most extensive cohort study of childhood cancer survivors, the Childhood Cancer Survivor Study, reported that the cumulative risk of subsequent neoplasm increased by 3.2% in 20 years and 9.3% in 30 years [17]. The mechanism by which secondary malignancy occurs is diverse. It is thought that ionizing radiation or the chemical agents used in chemotherapy increases the risk of SMN by causing DNA damage [18]. The types of cancer caused by SMN vary according to ethnicity, primary tumor distribution, and the types of chemotherapy or radiotherapy. In a multi-center retrospective study in Korean childhood cancer survivors, secondary malignancy occurred most frequently in the order of acute myeloid leukemia, myelodysplastic syndrome, thyroid cancer, and CNS tumor [19]. Among the 102 patients with SMN, 17 patients (17%) had primary CNS tumors. In this study that is targeting only primary CNS tumors, SMN distribution was similar to that of a Korean retrospective study (2 thyroid cancers, 1 acute myeloid leukemia, 1 acute B lymphoblastic leukemia, 1 diffuse large B cell lymphoma, and 1 meningioma).

Surveillance of SMN is crucial because there may be a risk of SMNs even after successful treatment of the primary tumor. In particular, long-term surveillance strategy is essential for SMNs because the risk increases even after 20 or 30 years [20]. Indeed, the patients included in this study also developed SMNs 10 years after completing therapy. The long-term management and surveillance of SMNs should be emphasized for the improvement of overall survival. Moreover, all patients with SMN were given radiation therapy to CSA. Given the good prognosis, efforts to reduce the area and dose of radiation therapy will be necessary.

There are several limitations to our study. First, this is a retrospective study and various types and doses of radiotherapy were applied, although homogeneous upfront chemotherapy was administered in a single institution. Because PRT was not available in our institution, any patient who desired to be treated with PRT had to visit another institution. Furthermore, there were also differences in follow-up duration between patients treated with XRT and PRT. Further studies with a larger patient cohort and a longer follow-up must be performed to compare the outcome and long-term complications between XRT and PRT.

## Conclusion

This study demonstrated that upfront chemotherapy using carboplatin, cyclophosphamide, etoposide and/or bleomycin according to the risk classification, along with radiotherapy, for the treatment of CNS GCTs showed good clinical outcomes and acceptable relapse incidence in our institution. However, late complications, including secondary malignancy, could substantially increase, and thus need to be thoroughly monitored. To reduce long-term complications, treatment of some HR patients, particularly those with germinoma but with high tumor markers, may be adjusted to a lower intensity.

## Data Availability

The datasets generated and analyzed during the current study are available from the corresponding author on reasonable request.

## Abbreviations

CNS GCTs: Central nervous system germ cell tumors
LR: Low risk
HR: High risk
NGGCTs: Non-germinomatous germ cell tumors
CSI: Craniospinal irradiation
EFS: Event-free survival
OS: Overall survival
AFP: α-fetoprotein
β-HCG: β-human chorionic gonadotropin
XRT: Photon radiation therapy
PRT: Proton radiation therapy
SMNs: Secondary malignant neoplasms
PFS: Progression-free survival
EVD: Extraventricular drainage
CSF: Cerebrospinal fluid
MRI: Magnetic resonance imaging
